# Under conditions of high wall shear stress, several PfEBA and PfRH ligands are important for malaria *Plasmodium falciparum* blood-stage growth

**DOI:** 10.1128/mbio.01499-25

**Published:** 2025-06-23

**Authors:** Emma Kals, Morten Kals, Pietro Cicuta, Julian C. Rayner

**Affiliations:** 1 Cambridge Institute for Medical Research, University of Cambridge 98524, Cambridge, United Kingdom; 2 Cavendish Laboratory, University of Cambridge 2152, Cambridge, United Kingdom; NIAID/NIH, Rockville, Maryland, USA

**Keywords:** malaria, *Plasmodium falciparum*, orbital shaker, growth assay, PfEBA, PfRH, bloodstream infections, host cell invasion

## Abstract

Malaria kills over 600,000 people annually, with all clinical symptoms arising from blood-stage infection. *Plasmodium falciparum* blood-stage replication happens primarily in the blood circulation, bone marrow, and spleen, where there are flow-generated forces, yet most *in vitro* growth assays are carried out in static conditions. We systematically tested the effect of orbital shaking on growth and linked it to the wall shear stress forces generated by the resultant fluid motion. Strikingly, there is a critical shaking speed, below which growth rates are reduced and above which growth increases. Forces at this critical speed correspond to previously measured forces in the microvasculature. Red blood cell invasion depends on two families of parasite attachment proteins, the *Plasmodium falciparum* erythrocyte-binding antigen and the *Plasmodium falciparum* reticulocyte-binding protein. Using a panel of knockouts, we show for the first time that several of these ligands have greater importance in high wall shear stress conditions, highlighting the importance of understanding the effect of fluid motion on parasite biology.

**IMPORTANCE:**

Malaria parasite growth occurs in dynamic environments like blood circulation, where fluid forces impact red blood cells and parasites. Yet, most laboratory growth assays are conducted in static environments, failing to replicate these forces. We explored the effects of growing the parasites on orbital shakers, which generate biologically relevant forces, and found that shaking speed critically impacts parasite growth, with reduced growth at speeds that mimic forces in the microvasculature. Importantly, using these conditions revealed invasion phenotypes not observed under static conditions. Understanding how fluid dynamics influence parasite growth offers a new approach to investigating malaria pathogenesis, with the potential to improve the development of therapeutic interventions.

## INTRODUCTION

All clinical symptoms of *Plasmodium falciparum* malaria are caused by the blood stage of the infection, during which the parasites invade and replicate inside red blood cells (RBCs). Blood-stage growth requires parasites inside mature infected RBCs, known as schizonts, to egress, releasing the merozoite form of the parasite that goes on to invade uninfected RBCs, a process that typically takes less than a minute (1). After invasion, the parasite develops into a new schizont containing 26–32 individual merozoites over the next 48 h (2, 3). Where in the body parasites develop and invasion occurs is not completely understood; postmortem analysis of patients with severe malaria has shown that there is a high attachment due to cytoadherence of infected RBCs to capillaries and postcapillary venules in organs (4), and so, a key site of invasion is likely in the subsequent vasculature. There is also increasing evidence that a significant proportion of parasite burden may be in the bone marrow (5) and spleen (6, 7). In these different environments, both parasites and RBCs will be exposed to shear forces resulting from gradients in the velocity, intrinsic to fluid flow in confinement. The conditions used in routine culture vary between laboratories, with some maintaining culture in static conditions and others maintaining cultures on an orbital shaker, particularly when higher parasite multiplication rates are needed, such as before transfection. However, the conditions used (including plate/flask size and volume, shaking platform, and orbital rotation speed) vary widely and are often not specified in papers, and while shaking is sometimes used for parasite maintenance, from the literature, it appears that most quantitative phenotyping assays such as for parasite growth, invasion, and drug resistance are still performed primarily under static conditions.

Shaking on an orbital rotation platform is a simple and low-cost method of introducing motion in parasite cultures. While it does not accurately mimic physiological conditions, orbital shaking creates shear forces comparable to those experienced in the vasculature and is commonly used when culturing epithelial cells (8). The fluid motion induced by shaking can be characterized by two fundamental flow regimes: laminar and turbulent. In laminar flow, fluid moves in smooth, orderly layers, creating shear forces that primarily arise from the interaction between the fluid and the container walls. In contrast, turbulent flow is characterized by chaotic, irregular motion with significant mixing between fluid layers, resulting in more complicated shear forces. Both flow types exert shear stress on the flask walls, referred to as wall shear stress (WSS), which will influence the interactions between parasites and RBCs in sedimented culture.

Several studies have reported that shaking influences the growth and behavior of *P. falciparum* in culture, though results have been inconsistent, likely due to varying methodologies and unquantified forces. Shaking has been shown to either enhance (9–13) or reduce growth rates (14). Shaking also distributes parasites more evenly through the culture, reducing the number of RBCs infected by multiple parasites (12, 14–16). However, the absence of systematic comparisons across a range of shaking speeds and flow regimes makes it challenging to untangle the precise effects of shear forces on parasite growth.

We therefore systematically investigated how changing experimental parameters impacted parasite growth under shaking conditions, with the aim of developing a simple, consistent assay format. Vessel geometry, fluid volume, fluid viscosity, orbital radius, and orbital rotation speed will all impact fluid motion and the resultant mechanical forces acting on both parasites and erythrocytes. By systematically testing a range of different conditions at different orbital shaking speeds, we show that growth under shaking is strongly impacted by shaking speed, vessel geometry, and hematocrit (HCT) and that shaking can both decrease and increase parasite growth. In doing so, we identified a critical shaking speed when the magnitude of the WSS is at its maximum and is comparable to that measured in the microvasculature, and parasite growth is decreased relative to static. We therefore applied this critical shaking speed assay to specifically explore the role of parasite attachment ligands under conditions of high physiological WSS. Parasite invasion of RBCs involves the *Plasmodium falciparum* erythrocyte-binding antigen (PfEBA) and *Plasmodium falciparum* reticulocyte-binding protein homolog (PfRH) families. Although individual deletions of these ligands do not block invasion (17, 18), except for PfRH5 (19, 20), their functional redundancy (21, 22) likely enables parasites to adapt to host immune responses and receptor variability (23–25). We have previously characterized the static growth rates of PfEBA and PfRH knockout lines and have shown that there are no significant growth rate changes compared to the control knockout lines, consistent with other studies (26). However, using the critical shaking speed assay, we demonstrate that several ligands play a much more important role under high shear stress conditions. This adds to our understanding of these pan-*Plasmodium* gene families and suggests that another reason for the large number of different PfEBA and PfRH ligands is to allow invasion under different shear stress conditions in different regions of the circulatory system.

## MATERIALS AND METHODS

### *Plasmodium falciparum* culture

*P. falciparum* strains (either the wild-type NF54, 3D7, or Dd2 lines or genetically modified lines) were cultured in human erythrocytes purchased from NHS Blood and Transplant, Cambridge, UK. Unless indicated, cultures were kept at a 4% HCT. Cultures were maintained in Roswell Park Memorial Institute (RPMI) 1640 media (Gibco, UK) supplemented with 5 g/L Albumax II, 2 g/L dextrose anhydrous EP, 5.96 g/L HEPES, 0.2 g/L sodium bicarbonate EP, and 0.05 g/L hypoxanthine dissolved in 2 M NaOH. Culture was performed at 37°C in a gas-controlled incubator or in a sealed culture container pre-gassed with a low-oxygen atmosphere of 1% O_2_, 3% CO_2_, and 96% N_2_ (BOC, Guildford, UK).

### Preparation of synchronized parasites for growth assay

All lines were cultured in static conditions before the assay. The lines were defrosted and sorbitol synchronized as described in references 26 and 27 and then delayed at room temperature to push the egress window to the intended start window. Smears were checked to confirm the culture was predominantly schizonts with few rings. All but 5 mL of the media was removed, and the infected blood was resuspended in the residual liquid, then layered over 5 mL of 70% Percoll in a 15 mL tube (Percoll Merck P4937, 10% 10× phosphate-buffered saline [PBS], 20% RPMI) and centrifuged at 1,450 relative centrifugal force (rcf) for 11 min with break set to 1 and accelerator to 3. The band of late-stage parasites at the Percoll-media interface was resuspended at a 4% HCT and incubated in a gas-controlled incubator at 37°C for 3 h. The Percoll separation was then repeated, but this time, the bottom pellet containing newly invaded rings was kept, and then sorbitol synchronized to eliminate any late-stage parasites, resulting in tightly synchronized parasites within a 3 h development window. The parasitemia was then counted using Giemsa smears, and the cultures were diluted to a 0.2%–0.5% parasitemia at a 4% HCT.

### Growth assay

Unless otherwise indicated, three replicates in the same blood for every condition were grown in parallel to provide technical replicates. Every two cycles, the samples were split into new blood (at least 50%), ensuring blood from three donors was tested. Every day ~3 h after the initial invasion window, the parasitemia was measured using flow cytometry. Five microliters of each well was diluted in 45 µL of PBS in a 96-well plate. The samples were stained using SYBR Green I (Invitrogen, Paisley, UK) at a 1:5,000 dilution for 45 min at 37°C. The samples were then run on an Attune NxT acoustic focusing cytometer (Invitrogen) with SYBR Green excited with a 488 nm laser (BL1-A) and detected by a 530/30 filter. The parasitemia was measured using the Attune NXT software. A plot of side scatter area vs forward scatter area (FSC-A) was used to gate for roughly the size of an RBC (Fig. S3a), and then singlets were gated using a forward scatter height vs FSC-A plot (Fig. S3b). Next, histograms for the BL1-A intensity were used to gate for the SYBR Green-positive RBCs. The percentage of positive RBCs represents the percentage of infected RBCs (Fig. S3c). Every 48 h, with the culture in ring stage, it was split to give 0.5% parasitemia.

### Shaking conditions

Shaking was performed at 45, 90, or 180 rpm using an orbital shaker (Celltron from Infors HT), which has a 25 mm shaking throw. Comparison of different culture vessels was performed at 4% HCT using a 50 mL polystyrene tissue culture-treated flask with an area of 25 cm^2^ (ref 353014 Falcon) with a 5 mL culture volume, a six-well plate with a 5 mL culture volume, a 24-well plate with a 1 mL culture volume, or a 96-well plate with a 100 µL culture volume. Different HCTs were compared in a six-well plate with a 5 mL culture volume.

### Determining the critical clustering transition for other culture format/shaking platforms

To recapitulate the inhibitory growth effects described here in other plate/well sizes or on other shaking platforms, it is necessary to first determine the minimum orbital rotation speed at which the blood in a well is kept in suspension, which we have termed the critical clustering transition. The well or culture vessel is set up with just blood and media, with the same hematocrit and volume as will be used for the assay. The vessel is placed on the shaking platform, and the blood is allowed to settle completely. A camera phone is mounted above the well on the shaking platform by taping it to a box placed above the well, so the whole well is in the field of view. Recording is started, and then the orbital rotation speed is gradually increased. When the speed is increased, the speed which has been set is said out loud. The speed is increased until there is a risk of the blood splashing out of the well. The video can then be played back, and from the audio, the first speed at which the blood starts to cluster in the center can be determined.

### Calculating growth

Growth was measured by calculating the parasite erythrocyte multiplication rate (PEMR). PEMR was defined in reference 28 as “The ratio between the number of parasites at any point in the intraerythrocytic development cycle (IDCn) and the number of rings in the IDCn +1.” Custom Python scripts were used for further analysis (available at 10.5281/zenodo.13372478). As shown previously (14, 29), starting parasitemia can affect growth rate demonstrated by the negative correlation seen when the parasitemia in the initial intraerythrocytic developmental cycle (IDCn) was plotted against the PEMR of the subsequent cycle (IDCn +1) (Fig. S4). The target starting parasitemia for all cycles was 0.5% (64.9% of measurements across all assays had a parasitemia of 0.25%–0.75%). The effect is most noticeable when the starting parasitemia is above 1%; therefore, any wells with IDCn of >1% were removed from the data sets (9.7% of measurements had an IDCn of >1% across all assays). Subsequent cycles were unaffected if parasitemia was correctly split to IDCn of <1%. Plots of the PEMR by cycle were manually assessed, and any data set was removed where very high parasitemia (IDCn > 4%) caused subsequent repeats to be very low (2.1% of measurements had an IDCn >4% across all assays). The multiple invasion rates were assessed in the ring-stage cultures using FlowJo (Tree Star, Ashland, Oregon) to plot BL1-A intensity, and the proportion of reads in peak was measured (Fig. S3).

## RESULTS

### Significant agitation is required to prevent RBCs from sedimenting

**Fig 1:**
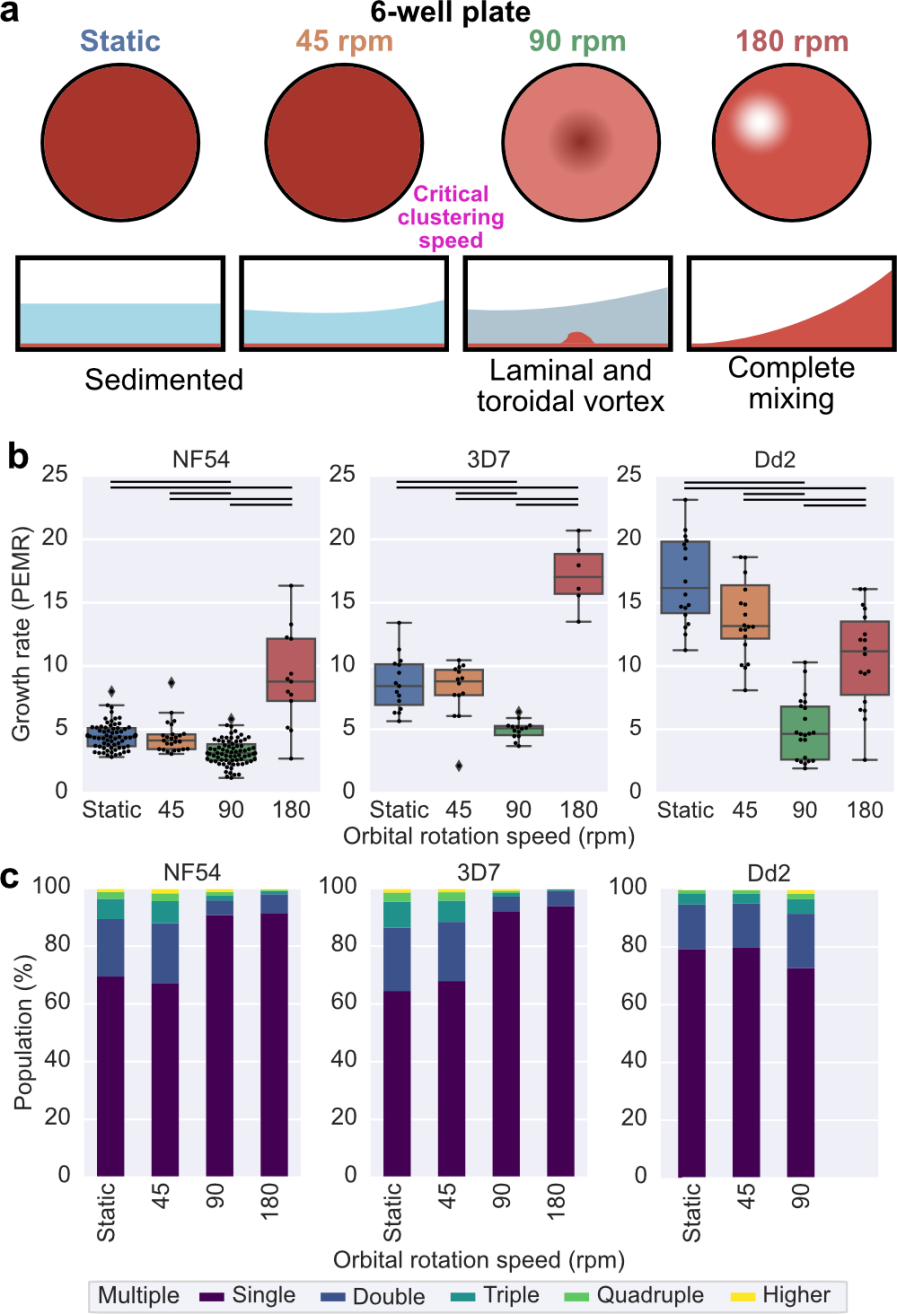
Shaking speed affects the growth rate of wild-type *P. falciparum* lines NF54, 3D7, and Dd2. All data were collected for samples cultured in a six-well plate, 5 mL culture volume, and a 4% HCT. (a) A schematic to represent visually what the motion of the blood looks like at the conditions for which growth was compared. In static conditions, the blood is sedimented at the base of the well; at 45 rpm, the fluid just starts to move over the sedimented blood; 90 rpm is just above the critical clustering speed, and the RBCs are largely confined to the vortex. At 180 rpm the conditions are highly turbulent, and the RBCs are completely mixed through the medium. The simulated mean WSS is shown for the conditions where the blood is still sedimented. (b) The growth rate is shown as the parasite erythrocyte multiplication rate (PEMR). The data displayed are from all experiments run under the indicated conditions across multiple assays. The lines between conditions indicate the significantly different conditions (*t*-test) at a greater than 5% significance level. (c) Graphs represent the mean frequency of different numbers of parasites in a single RBC for a given speed for each wild-type line. The frequency of single-infected, double-infected, triple-infected, quadruple-infected, and higher-infected rings is shown as a fraction of the total infected ring-stage parasites. The number of rings per erythrocyte was measured using the peak intensity of the ring-stage infected samples and the number of counts in each peak. Data sets without clearly defined peaks were excluded, which is why there are no data for Dd2 at 180 rpm.

We began by exploring fluid motion in standard malaria parasite culture conditions (six-well plate, 5 mL culture volume, 4% HCT). We filmed how the fluid motion of blood changed as the shaking speed increased (Movie S1 and Fig. S1). At low rotational speeds, the RBCs remain sedimented at the base of the well. As rotation speed increases, the media begins to move above the sedimented RBCs, which results in the cells being subject to the WSS. Then, as a critical threshold is surpassed, which we term the “critical clustering transition,” blood begins to cluster at the center of the well, forming a toroidal vortex of RBCs. Further speed increases cause a corresponding increase in the motion of the vortex structure, and more RBCs become suspended in the laminar flow region surrounding the central vortex. As orbital rotation speed increases, the RBCs gradually get mixed into the bulk media as the flow becomes more turbulent until all the RBCs are suspended (Fig. S2a). With this culture geometry, three shaking speeds can capture the different dynamic fluid conditions: 45 rpm, where the fluid is moving over the sedimented blood but the RBCs are not moving; 90 rpm, which is just above the critical clustering speed and the RBCs have largely moved into a central vortex but have not yet dispersed into the surrounding medium; and 180 rpm, where the conditions are highly turbulent and the RBCs have been suspended (Fig. 1a). Quantifying the WSS in these round wells under orbital motion is difficult because the fluid motion is non-uniform (30). To estimate WSS, we used the model presented in reference 8 and based on references 31 and 32. We simulated the mean WSS over time at three points along the bottom of the well (25%, 50%, and 75% between the wall and center) and reported the mean of these (Fig. S2b). WSS increases with orbital rotation speed. For the six-well plate, the mean WSS at the clustering transition speed (85 rpm) was simulated to be 2.7 N/m^2^, which interestingly is comparable to measurements of WSS in human microvessels (33), where late-stage parasites sequester (4).

### Shaking can both decrease and increase the growth rate of wild-type *P. falciparum* parasites

The growth of three wild-type *P. falciparum* strains (NF54, 3D7, and Dd2) was compared at static conditions and at 45 , 90, and 180 rpm (Fig. 1a). Growth was defined using the PEMR, as defined by reference 28, calculated by dividing parasitemia after invasion (ring stage) by parasitemia the day before (trophozoite/early-schizont stage). Parasitemia was measured using flow cytometry to calculate the percentage of DNA-positive (i.e., infected) RBCs (Fig. S3). The effect of starting parasitemia was carefully controlled (Fig. S4). The wild-type lines have different growth rates measured as PEMRs under static conditions. NF54, likely of West African origin (34), had a static PEMR of 4.2 ± 0.2 (Fig. 1b). 3D7 is a clone of NF54 (34) that has been cultured independently for 30 years and, while being genetically very similar (35), has multiple diverged phenotypes (36–38), including those shown here in growth rate under static conditions, with a PEMR of 8.7 ± 0.6 (Fig. 1b). Dd2 is a strain of Southeast Asian origin chosen as it has many invasion characteristics distinct from 3D7/NF54 and has the highest growth rate in static conditions with a PEMR of 13.7 ± 0.5 (Fig. 1b). These measurements are comparable with previous measurements (39). There was no significant difference in PEMR between static conditions and 45 rpm for all lines, where the RBCs are not suspended. At 90 rpm, just above the critical clustering speed, the growth rate drops for all lines, while at 180 rpm, the growth rate increases for 3D7 and NF54 and decreases relative to static for Dd2. This shows that shaking does not always produce better growth/invasion rates and that the relationship between growth and shaking is complex (Fig. 1b).

We quantified the degree to which erythrocytes were infected with multiple parasites based on peaks in the intensity of DNA staining in the ring-stage cultures as described previously in reference 40 (Fig. S3d). We refer to the number of rings measured in each RBC as its multiplicity of infection (MOI) (41). Previous observations using Giemsa smears have shown that up to five rings can be observed in a single RBC and that different strains have different mean MOI rates (41). For 3D7 and NF54, there was no difference in the proportion of RBCs with two or more rings between static conditions and 45 rpm but much lower rates at both 90 and 180 rpm (Fig. 1c), in keeping with previous reports of lower rates of multiple infected rings when culturing under shaking conditions (11, 12, 15, 16). This trend was not observed for Dd2 (Fig. 1c), but the MOI data for Dd2 is less reliable as its replication cycle is shorter.

### The critical clustering speed correlates to a drop in growth rate across different culture conditions

**Fig 2:**
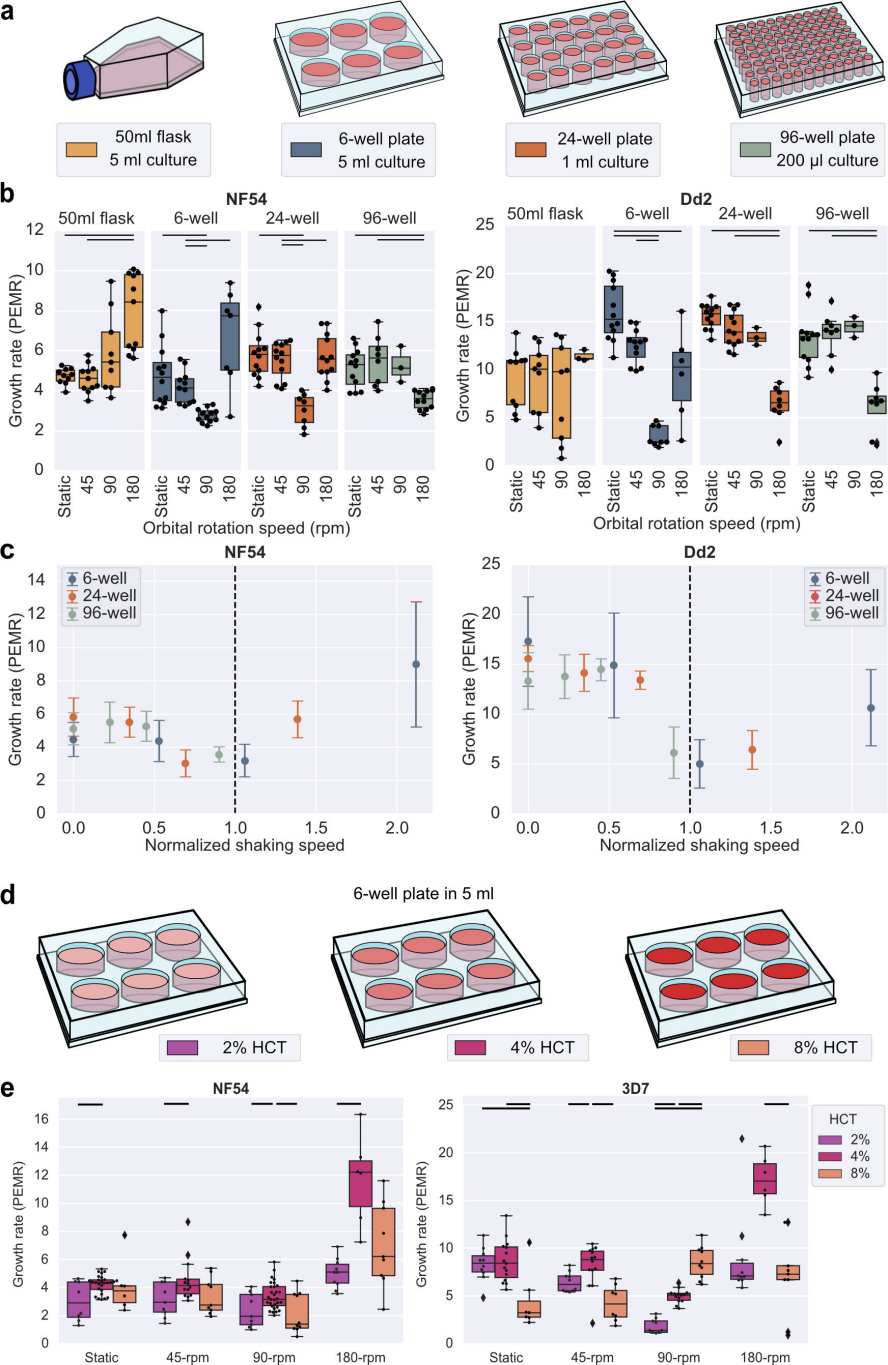
The type of culture container and hematocrit affects the growth rate of wild-type lines of *P. falciparum*. (a) Schematics illustrate the different cultural conditions that were compared. (b) The wild-type lines NF54, 3D7, and Dd2 were tested. All cultures were kept at a 4% HCT. The mean growth rate is shown as the parasite erythrocyte multiplication rate (PEMR). The experiment was performed in triplicate technical repeats; each point shows a PEMR measured for a single invasion cycle for an individual well. The lines between conditions indicate the significantly different conditions (*t*-test or rank sum) at a greater than 5% level of significance. (c) Growth rates from panel b for the round wells plotted against the normalized shaking speed. The shaking speed was normalized to the rotation speed at which the blood was first seen to cluster at the center of the well (Fig. S2a). (d) Schematics illustrate the different hematocrit conditions that were compared. (e) All cultures were kept in a 5 mL culture volume in a six-well plate. All data presented in the figure were collected in parallel. The mean growth rate is shown as the PEMR; each point shows a PEMR measured for a single invasion cycle for an individual well. The lines between conditions indicate the conditions that were significantly different (*t*-test) at a greater than 5% level of significance.

Next, we utilized the changing vessel shape and size to change the resultant fluid flow produced at a given speed (Fig. 2a). Filming the changes in the blood motion across shaking speeds showed a consistent fluid flow change as the rotational speed was increased (Movie S2-S4 and Fig. S5). However, the smaller the well, the higher the orbital rotation speed required for the critical clustering transition. This pattern was observed for all vessels, but the fluid flow was more irregular in the culture flask due to its non-uniform shape, leading to more fluid mixing even at very low shaking speeds. We tested the growth in the different vessels of wild-type lines NF54 and Dd2 at the same range of shaking speeds tested previously (Fig. 2b).

The critical clustering transition speed (when blood began to cluster at the center of the well; see Fig. S2a) provided a way to normalize the shaking speeds across round well sizes. When the mean growth rates were plotted relative to this normalized orbital rotation speed, there was a clear pattern for both wild-type NF54 and Dd2, with the dip in growth rate occurring close to the clustering transition speed and growth increasing once normalized rotation increases beyond this transition (Fig. 2c). Interestingly, simulating WSS for the different well types shows that the shear forces at the critical clustering transition speed are similar in magnitude (Fig. S2b). This is likely because the shear stress is a determining factor in suspending the RBCs. Thus, normalizing to the critical clustering speed will, to some degree, normalize the shear forces experienced by the parasites. As expected, the culture flask/volume also changed how a given shaking speed affects the multiple invasion rates (Fig. S6a), and for NF54 and 3D7 but not Dd2, the transition between high to low percentage of multiple infected erythrocytes happens at the normalized critical shaking speed (Fig. S6b). Another variable likely affecting how shaking impacts growth is the erythrocyte density in the culture (hematocrit). It is difficult to measure hematocrit in the human body; experimental measurements done in a cat measured hematocrits from ~45% HCT in large arteries and veins down to ~4% HCT in capillaries (42), and human hematocrits are assumed to be similar. The growth of 3D7 and NF54 was compared at 2%, 4%, and 8% HCT (Fig. 2d and e). Altering hematocrit changes how a given shaking speed affects growth but not the multiple invasion rate (Fig. S7).

Hematocrit will affect the frequency of contact between merozoites and erythrocytes during the window in which the merozoites are invasion competent after egress, but it will also affect the density of parasites at a given HCT; a parasitemia of 0.5% will result in twice the density of parasites per culture volume in 4% HCT vs 2% HCT and four times the density in 8% HCT vs 2% HCT. This is one factor that limits testing of higher HCT as growth is adversely affected by high parasitemia in an *in vitro* setting (Fig. S4). However, in general, comparing a single hematocrit as shaking speed increases is consistent with the pattern of decreased growth followed by increased growth (Fig. 2e), demonstrating the importance of keeping hematocrit consistent when comparing growth rates under shaking conditions.

### Deletion of PfEBA and PfRH invasion ligands changes the impact of shaking on growth

We hypothesized that high WSS leads to lower growth rates because it makes it harder for merozoites to attach to RBCs during invasion. The PfEBA and PfRH family of ligands has been linked to the initial steps of invasion (43), and we have recently shown that several members of these families are key determinants of the strength of attachment between merozoites and RBCs (26). They are largely thought to be functionally interchangeable. We were therefore interested in whether disruption of PfRH and PfEBA proteins would impact the change in growth between static and shaking conditions at the critical transition point (six-well plate at 4% HCT and 90 rpm). A panel of PfRH and PfEBA knockout lines in the NF54 background was tested, along with two control knockout lines constructed in the same manner (26). The control lines have the gametocyte-specific proteins PfP230P or Pfs25 knocked out, which have no known role in invasion (44, 45).

**Fig 3:**
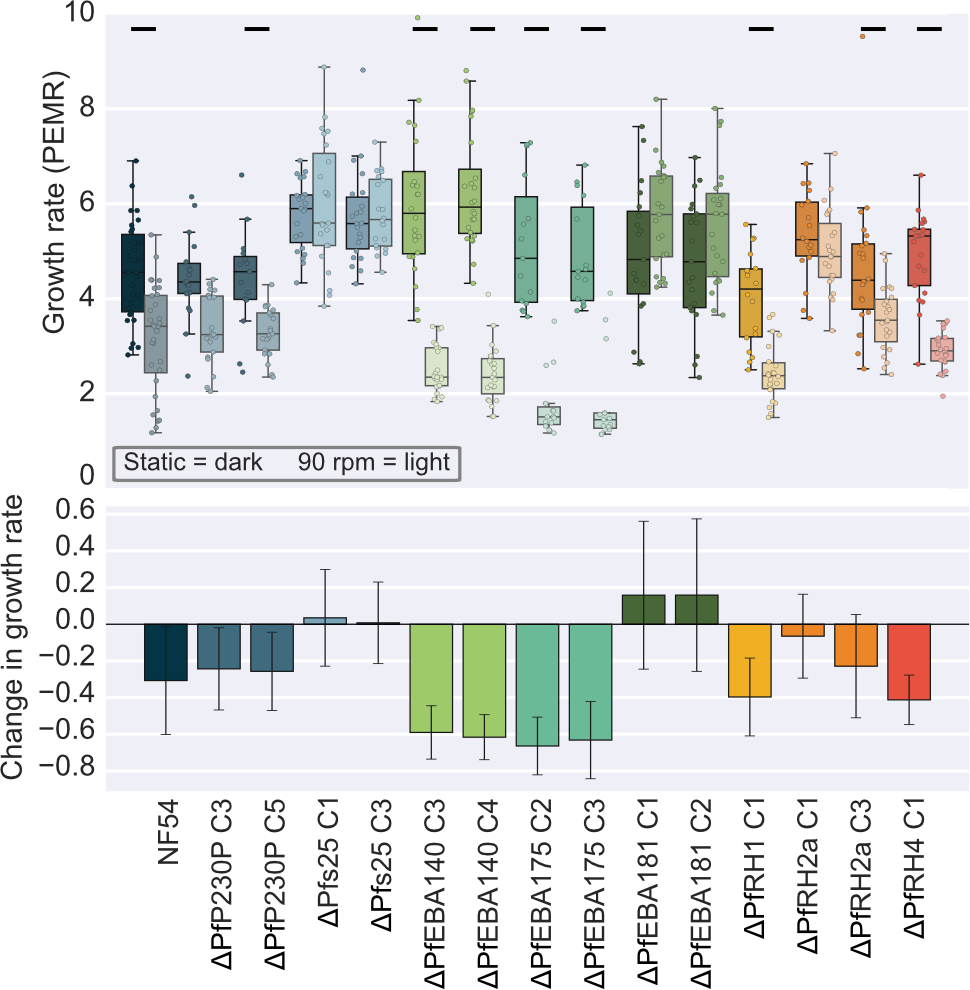
Knocking out specific invasion ligands shows flow-dependent effects. Experiments were carried out to compare PfEBA and PfRH knockout lines constructed in the NF54 background growth rates under static and detrimental shaking conditions at 90 rpm; two clones of each line were tested wherever possible. All cultures were grown at 4% HCT in 5 mL in a six-well plate. The top box plot shows the mean parasite erythrocyte multiplication rate (PEMR) of the knockout lines. The central red line shows the median, with the top and bottom of the box at the 25th and 75th percentiles and the whiskers showing the total range of the data. The bottom bar plot shows the relative difference in growth in shaking compared to static for each line. The error bars show the standard deviation. The lines between conditions indicate the significantly different conditions (*t*-test or rank sum) at a greater than 5% level of significance.

Where possible, two clones (cX and cY) were tested to assess the variability between clones. The data for the static growth rates of these lines were previously published (26), which showed similarly to what others have previously reported (22, 23, 46–49), that deletion of these ligands had little impact on growth under static conditions (Fig. 3). There were some minor differences in growth relative to wild-type NF54, likely due to epigenetic differences acquired in growth rates of clones during the extended period of culture required to generate transgenic parasites, as has been reported previously (29, 50, 51). Several of the lines showed higher rates of multiply infected erythrocytes than NF54, including ∆Pfs25c1/3, ∆PfEBA181c1/2, ∆PfRH2ac1 (but not c3), and ∆PfRH4c1 (Fig. S8).

When PEMR was compared between growth in static conditions and shaking at the critical clustering transition point for NF54 and control lines (∆PfP230P and ∆Pfs25), there was little (less than 30%) or no significant difference (Fig. 3), as shown previously (26). By contrast, when compared under shaking conditions, several PfEBA and PfRH knockouts relative to NF54 lines had a significantly lower PEMR: ∆PfEBA140c3/c4 (c3 2.4-fold lower *P* ≤ 0.0000, c4 2.6-fold lower *P* ≤ 0.0000), ∆PfEBA175c2/c3 (c2 3.0-fold lower *P* = 0.0003, c3 2.5-fold lower *P* = 0.0013), ∆PfRH1c1 (1.7-fold lower *P* = 0.0009), and ∆PfRH4c1 (1.7-fold lower *P* ≤ 0.0000) (Fig. 3). It is interesting that for the ∆PfEBA181c1/c2, while there was no significant difference in PEMR between static conditions and 90 rpm, at 90 rpm, the PEMR for ∆PfEBA181c1/c2 was significantly higher than NF54, which might indicate that reduced PFEBA181 aids in growth under these shaking conditions. This suggests that growth under shaking at the critical transition point can reveal growth/invasion phenotypes that are not detectable through static growth assays. In addition, the loss of several PfEBA and PfRH proteins (PfEBA140, PfEBA175, PfRH1, and PfRH4) seems to be more detrimental to growth under these shaking conditions, meaning these proteins may be more important to binding in conditions of high WSS, which in this assay set up are similar to the conditions that schizonts will experience in the microvasculature.

### Changes in growth rate are predominantly due to changes in invasion

**Fig 4:**
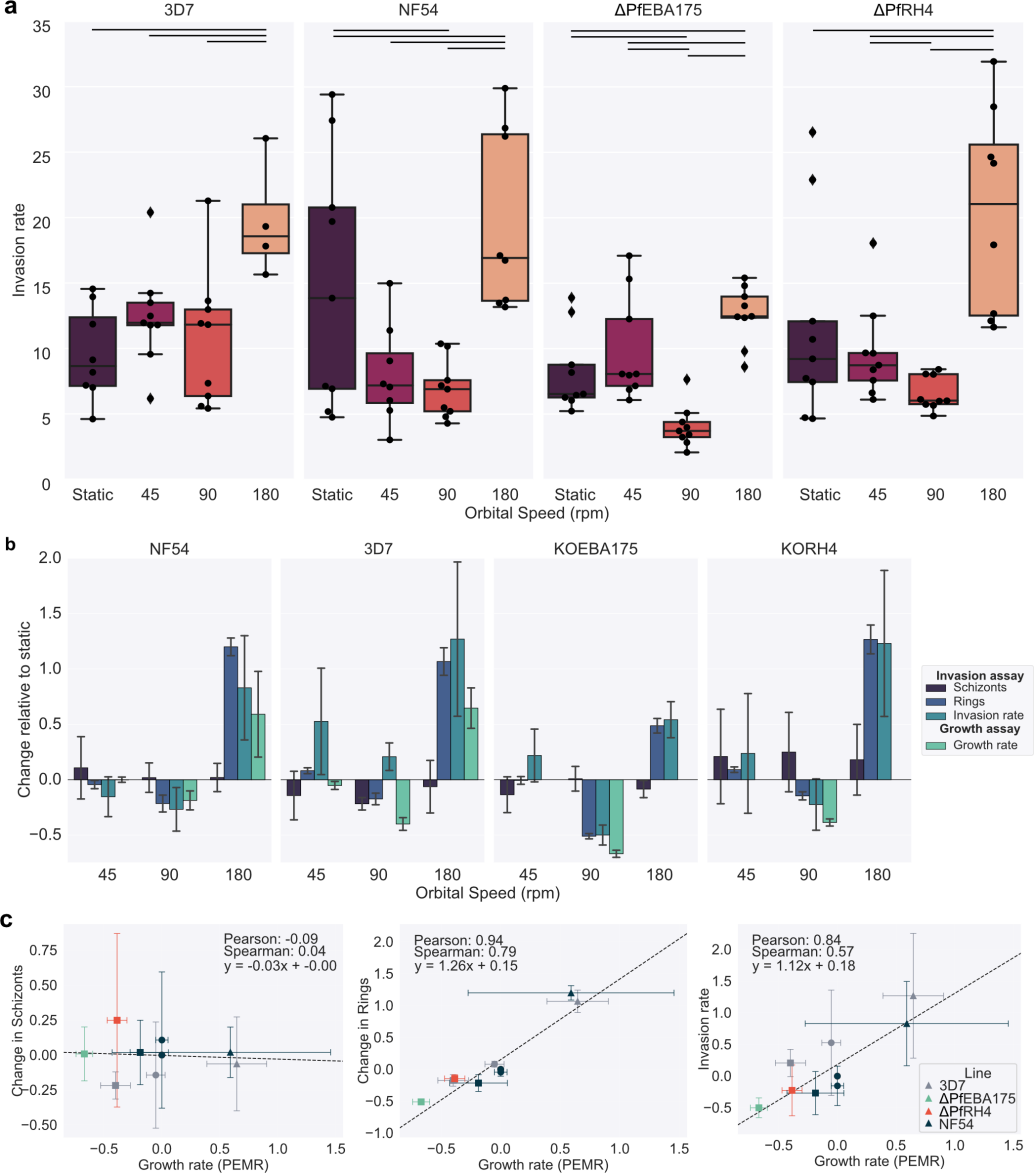
Evaluating if changes in growth rate are due to changes in invasion rate. All cultures were kept at a 4% HCT in a 5 mL culture in a six-well plate. All data presented in the figure were collected in parallel. Wild-type lines 3D7 and NF54 were tested along with ∆PfEBA175c2 and ∆PfRH4c1, which are in the NF54 background. Measurements were taken before and after a 3.5 h incubation window. The percentage of rings and schizonts was measured for each well before and after the invasion window. The change in newly invaded RBC rings was calculated by subtracting the percentage of rings present before incubation from the percentage of rings measured after invasion. The change in schizonts was calculated by subtracting the percentage of schizonts present after incubation from the percentage of rings measured before invasion. The invasion rate was calculated by dividing the change in the rings by the change in schizonts. Anything with an invasion rate of about 35 was excluded as an outlier, as there is a maximum of 32 merozoites per schizont. (a) Box plot comparing the invasion rates measured for the different lines across the shaking speed; each point is a single invasion cycle for an individual well. The lines between conditions indicate the conditions that were significantly different (*t*-test) at a greater than 5% level of significance. (b) The change in schizonts, change in rings, and invasion rate were compared to the previous growth rate measurements. All measurements were normalized to the static measurement to allow comparison. No data were collected for the growth rate of the knockout lines at 45 or 190 rpm. (c) Scatter plot comparing the normalized growth rates to the normalized change in schizonts, change in rings, and invasion rate.

Finally, we tested whether the changes in growth are due to the effects of shaking on invasion rather than another point in the life cycle. Schizonts were isolated from a tightly synchronized culture, resuspended at 4% HCT, and then split across the wells of six-well plates. The plates were then subjected to either static or shaking conditions for 3.5 h to allow for invasion to occur (Fig. 4a). Invasion rates showed the same pattern as growth rates, decreasing at 90 rpm and increasing at 180 rpm. The knockout lines tested ∆PfEBA175c2 was significantly different from NF54 at 90 rpm (*P* = 0.0065) and 180 rpm (*P* = 0.0101) but not at static conditions or 45 rpm. Whereas ∆PfRH4c1 was not significantly different from NF54 at any speed (Fig. 4a). To allow better comparison between conditions, results were normalized to static (Fig. 4b). There was no significant difference in the change in schizonts between conditions and no correlation between the change in schizonts and the corresponding growth rate (Pearson correlation coefficient −0.09) (Fig. 4c), meaning the changes in invasion rate are not due to shaking affecting the ability of parasites to egress from the schizonts. However, the change in rings and invasion rate correlates highly with growth (Pearson correlation coefficients 0.94 and 0.84, respectively) (Fig. 4c), for all lines and conditions tested, indicating the effect of shaking on growth is predominantly due to its impact on invasion.

## DISCUSSION

**Fig 5:**
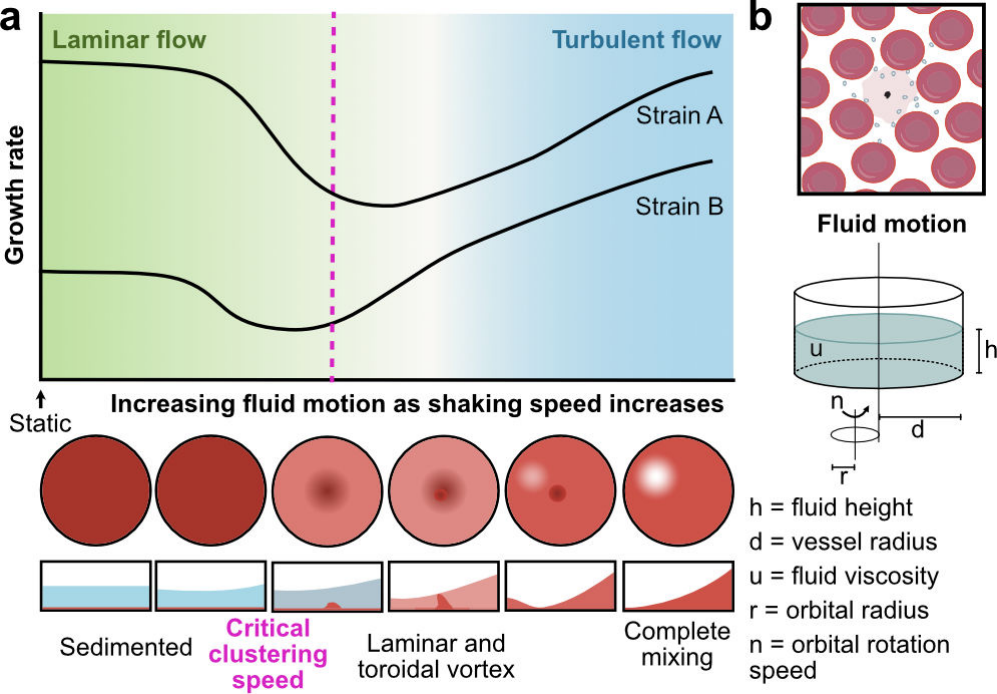
Summary of effects of shaking on *P. falciparum* growth. (a) Cartoon of the relation we have shown between increased orbital rotation speed and growth in round wells. Alongside are the observed changes in fluid motion. (b) Summary of the factors determining fluid motion when a round well is on an orbital shaker.

We have shown for the first time that there is an interesting behavior of decreased parasite growth at low orbital rotation speeds, in contrast to previous reports of increased growth under higher speeds (Fig. 5a). While unexpected, the fluid mechanics can explain these changes. Observation of the changes in RBC behavior showed that different phases can be distinguished as shaking speeds increase (Fig. 1a and Fig. S1). The speed at which the RBCs transition from sedimented to beginning to cluster at the center of the well, which differs, depending on vessel size/geometry and culture volume (Fig. 5b), represents a point at which the fluid motion will exert high shear stress on the sedimented blood. Strikingly, regardless of strain or vessel used, this point resulted in the lowest growth rate (Fig. 2c). The fact that changes in growth rate at different shaking speeds are mirrored by changes in invasion rate at those speeds strongly suggests that this dip in growth occurs because the high WSS at this transition point reduces the rate of successful attachments between merozoites and RBCs. In addition, disruption of several *P. falciparum* ligands known to be involved in attachment (PfEBAs and PfRHs) further reduces growth rates at this critical transition speed (Fig. 3). At higher shaking speeds, when the blood becomes suspended and the conditions become more turbulent, growth rates increase and the MOI rate falls (Fig. 5a and 2c and Fig. S6b). These turbulent conditions likely increase the frequency of contact between merozoites and RBCs, which overcomes the detrimental effect due to the increased WSS. We have previously shown that different wild-type lines have different attachment frequencies and detachment forces using an optical tweezer-based assay (26), potentially one reason why different lines are affected to different degrees (Fig. 1 and 5) and existing literature (9, 13, 52).

*P. falciparum* grows differently at different orbital speeds (Fig. 1), in different flasks (Fig. 2a), and over different hematocrits (Fig. 2e). We have presented a clear framework to design future experiments that can be adapted to a specific laboratory setup, as it is easy to visually determine the lowest orbital rotation speed at which blood starts to cluster in a round, flat-bottom well plate. This provides an easy way of determining conditions likely to produce either detrimental (at the critical clustering point) or favorable (above this transition point) conditions. The method presented here also provides a new and simple phenotyping assay to assess factors important to growth and invasion under physiologically relevant shear force conditions. For a 5 mL culture in six-well plates filled with 4% HCT culture and shaken at 90 rpm, the mean shear was 2.5 N/m^2^, while WSS will vary across the vessels of the circulation system. This is similar to the mean WSS measured in human conjunctival capillaries at 1.5 N/m^2^ (33). As schizonts have been shown to sequester in the microvasculature (4), this means that the forces experienced by parasites at the critical shaking speed are in the physiological range.

Using this new phenotyping assay in combination with PfEBA and PfRH knockout lines, we have established that the invasion ligands PfEBA140, PfEBA175, PfRH1, and PfRH4 are more important for growth under conditions of high shear stress than in static conditions (Fig. 3) where none of these lines had growth defects relative to controls (26). In the case of PfRH4, this fits with the previous observation that PfRH4 expression is upregulated in some lines when they are grown under constant shaking conditions (13, 52). In addition, when the attachment characteristics of the same panel of PfEBA and PfRH knockout lines were assessed using an optical tweezer-based assay, the only line in which there was a significantly decreased detachment force was ∆PfRH4 (53). The specific phenotype of decreased invasion in the absence of PfRH4 has not previously been observed nor have the other ligands been linked to invasion under shaking conditions. It is currently thought that the interactions of PfRHs and PfEBAs during invasion are largely redundant, and these data provide evidence that several proteins may actually be more crucial under high shear forces similar to those experienced *in vivo*. An area for future investigation is whether different ligands could be utilized in different environments in the body, which motivates the further development of a system where invasion can be quantified under a more uniform flow. It would also be interesting to perform assays where other factors such as ligand availability on RBCs are varied, such as by testing the effect of shaking on invasion into reticulocytes.

## Data Availability

All code, raw data, graphs, and videos supporting the findings of this study are available in the Zenodo repository at 10.5281/zenodo.13372478.

## SUPPLEMENTAL MATERIAL

Supplemental material mbio.01499-25-s0001.pdf Supplemental figures and tables; links to supplemental movies.

ASM does not own the copyrights to Supplemental Material that may be linked to, or accessed through, an article. The authors have granted ASM a non-exclusive, world-wide license to publish the Supplemental Material files. Please contact the corresponding author directly for reuse.
