# Supplementary material for: Under conditions of high wall shear stress, several PfEBA and PfRH ligands are important for malaria Plasmodium falciparum blood-stage growth
Source: mBio. 2025 Jun 23;16(8):e01499-25. doi: 10.1128/mbio.01499-25 (PMC12345213; doi:10.1128/mbio.01499-25)
Supplement: Supplemental material — Supplemental figures and tables; links to supplemental movies. [file mbio.01499-25-s0001.pdf]

Supporting Information for

Growing Malaria Parasites at a Critical Shaking Speed Mimicking Physiological Flow Reveals New Phenotypes for Invasion Ligands

Emma Kals, Morten Kals, Pietro Cicuta, Julian C. Rayner

Julian C. Rayner.
E-mail: jcr1003@cam.ac.uk

This PDF file include**s:**

Figs. S1 to S8
Legends for Movies S1 to S4
SI References

Other supporting materials for this manuscript include the following:

Movies S1 to S4

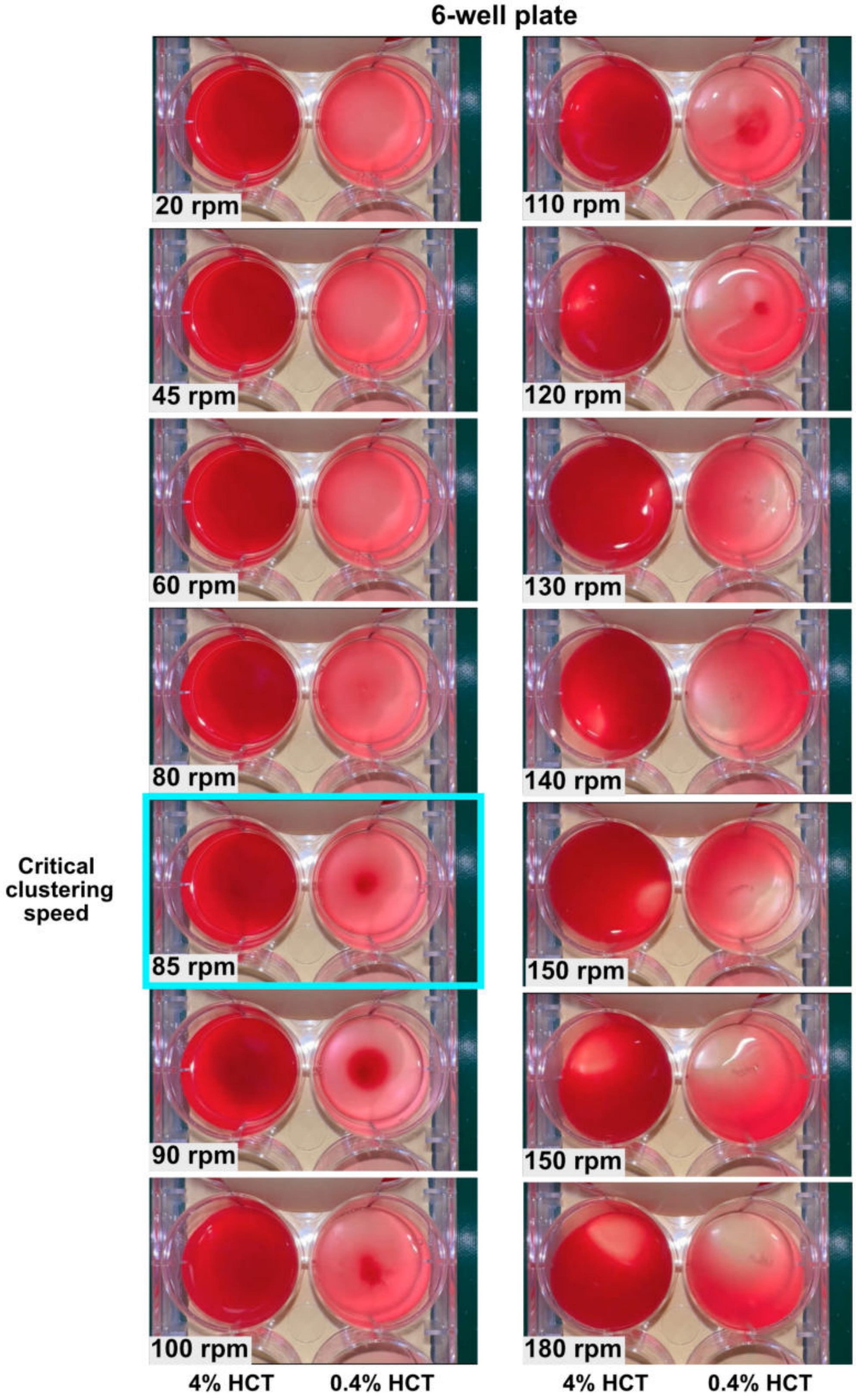

Fig. S1. Comparison of the effect of different shaking speeds on the suspension of blood in culture. Data is shown for 6-well flat-bottomed well plates with 5 ml culture volume at 4% (left well) and 0.4% HTC (right well). Images show how the blood goes from sedimented uniformly at low speeds, to clustering in the center, to being uniformly suspended in the culture. The blue outline represents the critical clustering speed where clear clustering starts to occur.

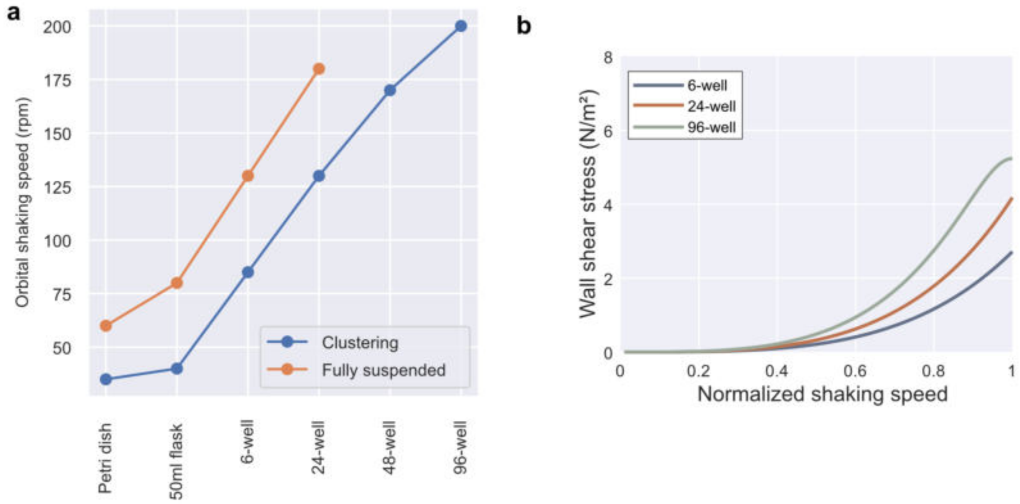

Fig. S2. Characterizing fluid motion (a) Shows the orbital shaking speed at which the RBCs start to cluster in the centre of the well and at which the RBCs are fully suspended in the bulk medium. The conditiosn tested were a 100 mm petri tissue culture dish (ref 353003) with a 10ml culture volume, a 50ml Polystyrene tissue culture treated flask which had an area of 25cm^2 (ref 353014 Falcon) with a 5ml culture volume, a 6-well plate with a 5ml culture volume, a 24-well plate with a 1ml culture volume, a 48-well plate with a 1ml culture volume or a 96-well plate with a 100μl culture volume. Our orbital shaking platform has a 25mm shaking throw and all samples were at a 4% HCT. For the 48 and 96 well plates, full suspension does not occur below 200 rpm, which was the top speed of our shakers. **(b)** Simulating wall shear stress (WSS) for each plate size at the volumes used in our experiments using the PT-Stokes model from (1) shows that the shear forces experienced in each plate type are of the same order of magnitude at the clustering speed threshold. Shaking speed normalized to clustering speed for each plate well size.

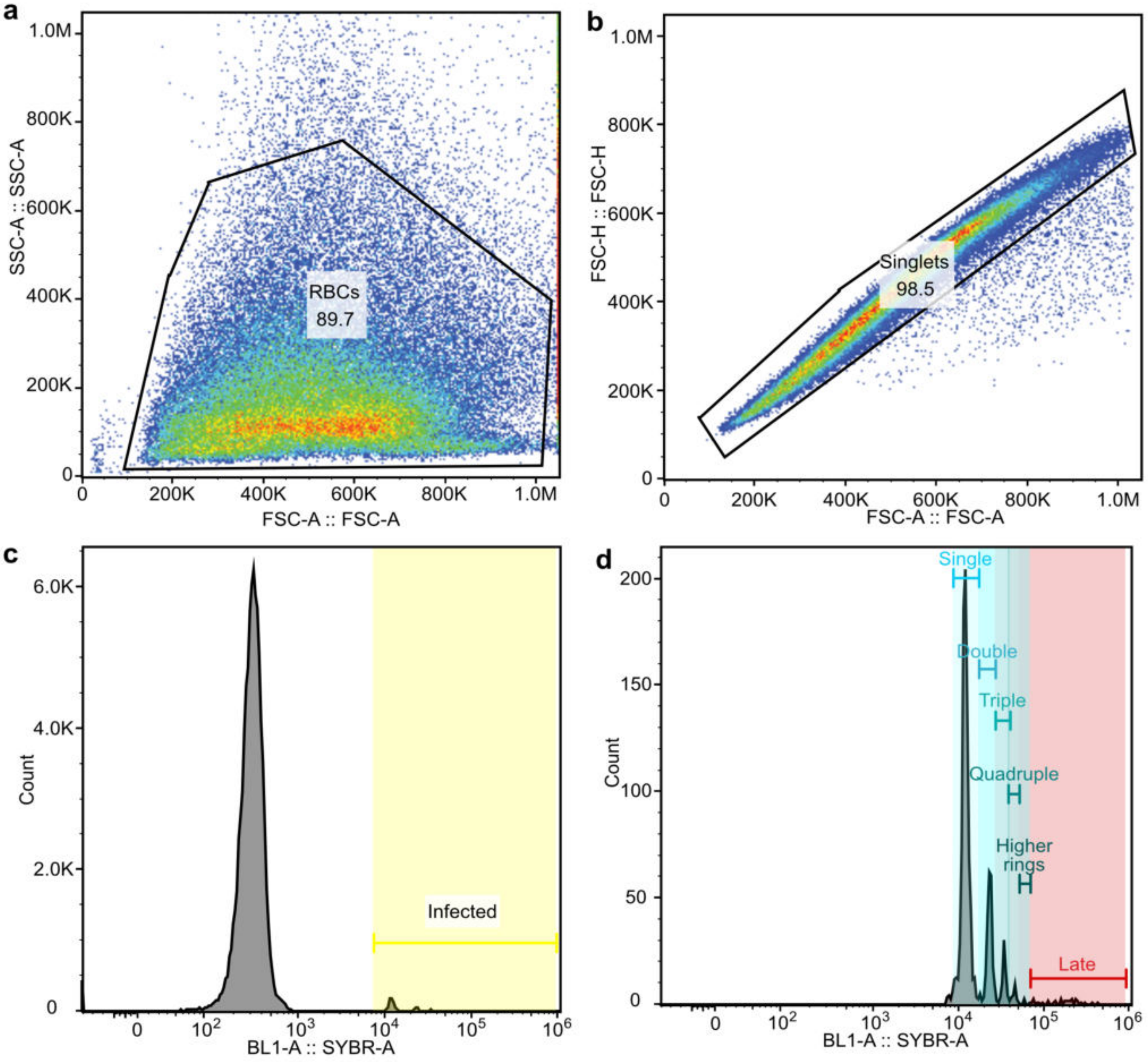

Fig. S3. Gating used to determine parasitemia and rate of multiple infections based on flow cytometry. Gating steps were taken to measure multiple invasion rates during the growth assay. The graphs shown are from one well on day 5 from NF54 in static condition for the growth assay that compared the PfEBA and PfRH knockouts. Analysis was done in FlowJo. The gates shown here were applied to all the data. **(a)** SCC-A vs FSC-A was plotted, and a gate was drawn to select for particles detected of roughly the right size for erythrocytes. **(b)** FSC-H vs FSC-A was plotted, and a gate was drawn to select the predominantly singlet population to exclude any signal from two or more erythrocytes clustered together. **(c)** A histogram of the BL1-A laser intensity was plotted, which detected the SYBR Green staining of the infected erythrocytes. A gate was set based on the signal of unlabelled erythrocytes. Anything with a high signal was defined as infected. Parasitemia was determined as the percentage of counts in this region out of the total number of counts after the initial two gates were applied. **(d)**Finally, for the samples collected when the parasites were early rings, additional gates were used in the region defined as infected for the peaks which represented single or multiple infected erythrocytes. This allowed calculation of the fraction of infected erythrocytes that had single, double, triple, quadruple, or higher infections and the even higher intensity region which represented any late-stage trophozoites or schizonts present.

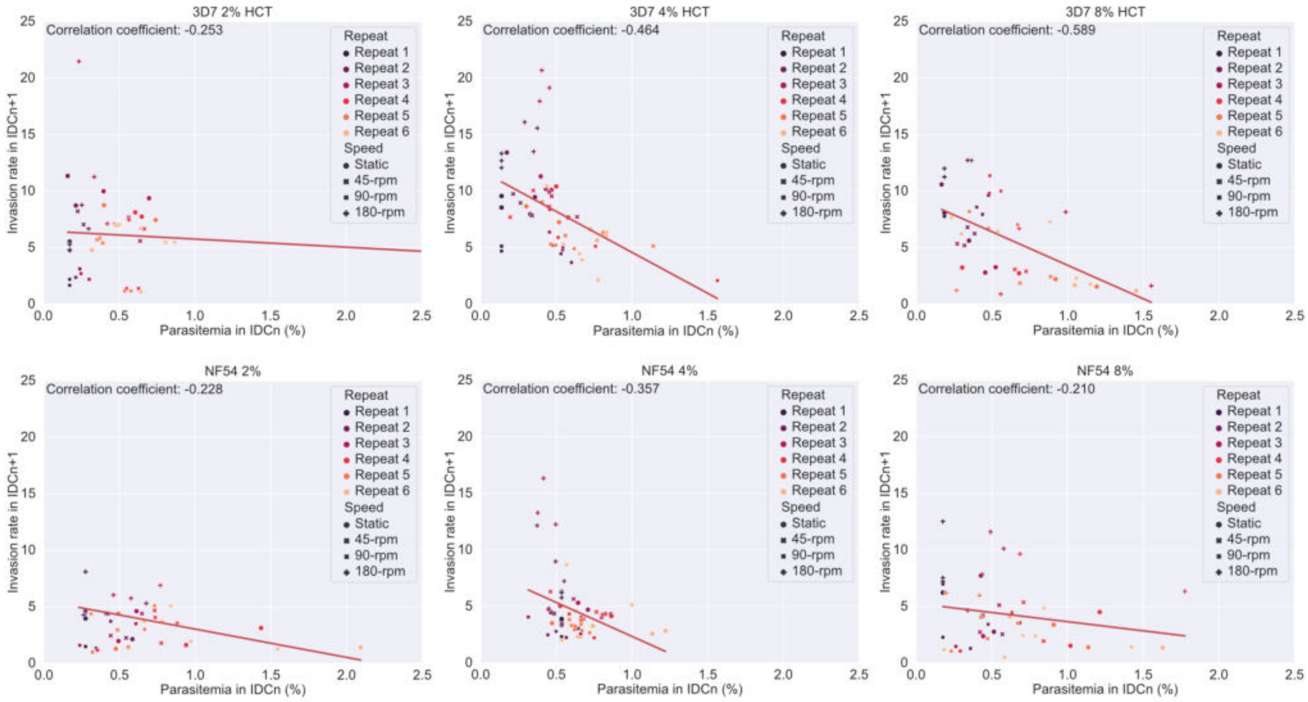

Fig. S4. The effect of the starting parasitemia on invasion rate. Plotted is the impact of the parasitemia in the initial intra-erythrocytic developmental cycle (IDCn) on the invasion rate of the subsequent cycle (IDCn+1). The data shown is for all NF54 and 3D7 data sets in a single assay in which the effect of different haematocrits was tested. Repeats were collected over different invasion cycles, as shown by different colours, and the different shaking speeds as shown with different symbols.

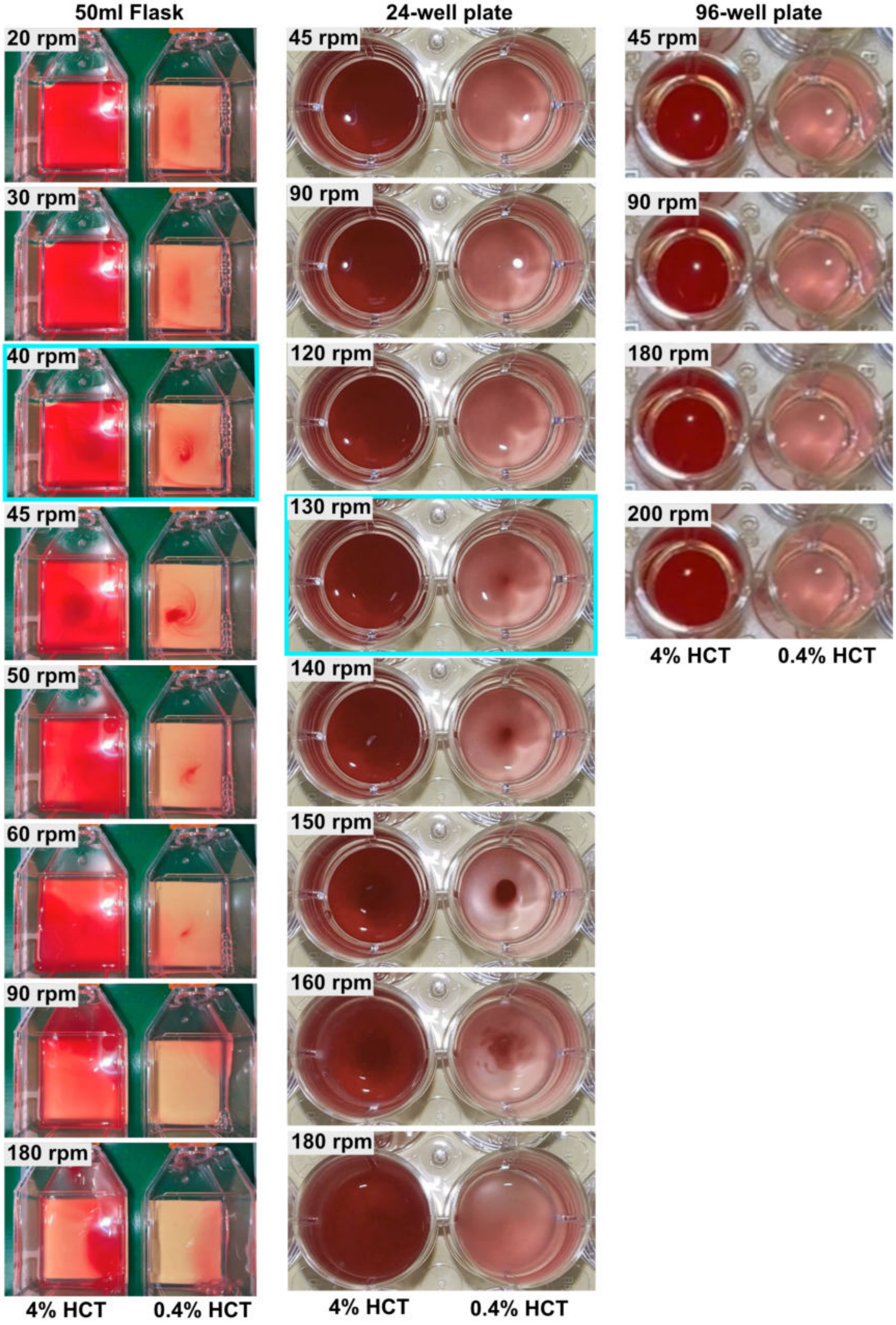

Fig. S5. Comparison of the effect of different shaking speeds on the suspension of blood in culture in different culture vessels. Data is shown for a 50 ml culture flask with a 5 ml culture volume, a 24-well flat-bottomed well plate with 1 ml culture volume and 96-well flat-bottomed well plate with 100 μl culture volume. All are at 4% (left well) and 0.4% HTC (right well). Images show how the blood goes from sedimented uniformly at low speeds to clustering in the center to being uniformly suspended in the culture. The blue outline represents the critical clustering speed where clear clustering starts to occur.

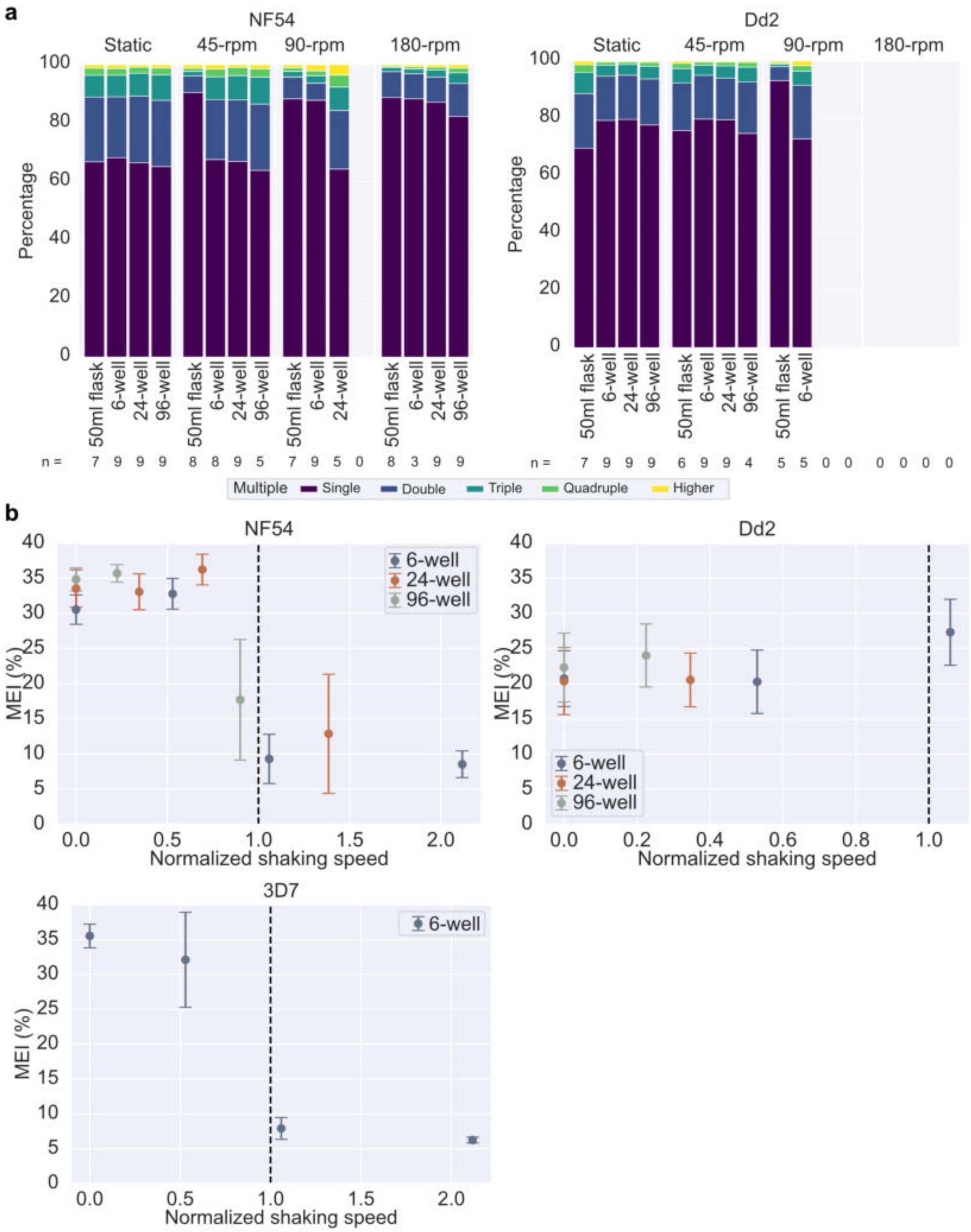

Fig. S6. Comparison of the effect of different culture containers and culture volumes on the impact of shaking speed on multiple invasion rates. All cultures were kept at a 4% HCT. All data presented in the figure was collected in parallel. This is the data for the same assay as in Fig 3. **(a)** Graphs representing the mean frequency of different multiple erythrocyte infections for a given speed for each wild-type line. The frequency of single-infected, double-infected, triple-infected, quadruple-infected, and higher-infected rings is shown as a fraction of the total infected ring-stage parasites. The number of rings per erythrocyte was measured using the peak intensity of the ring-stage infected samples by counting the fraction of counts in each peak. **(b)** Multiple erythrocyte infection (MEI) as a percentage of overall invasions relative to orbital shaking speed for NF54, 3D7 and Dd2. Shaking speed normalized to clustering threshold for the 6-well plates.

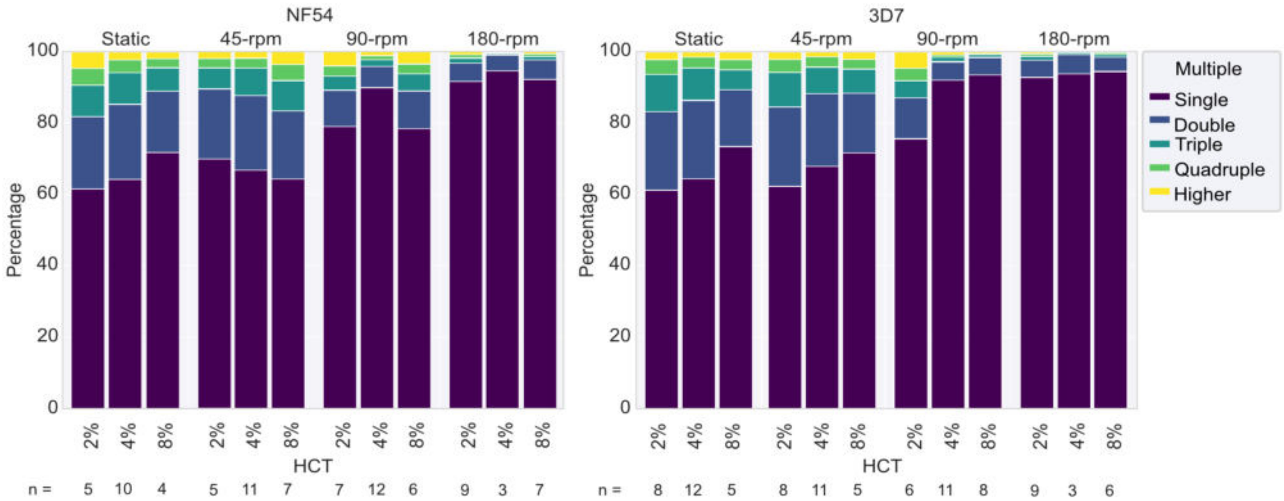

Fig. S7. Comparison of the effect of different haematocrit on how shaking speed affects the multiple invasion rates of NF54 and 3D7. All cultures were kept in a 5 ml culture volume in a 6-well plate. All data presented in the figure was collected in parallel. Graphs representing the mean frequency of different multiple erythrocyte infections for a given speed for each wild-type line. The frequency of single-infected, double-infected, triple-infected, quadruple-infected, and higher-infected rings is shown as a fraction of the total infected ring-stage parasites. The number of rings per erythrocyte was measured using the peak intensity of the ring-stage infected samples and the number of counts in each peak.

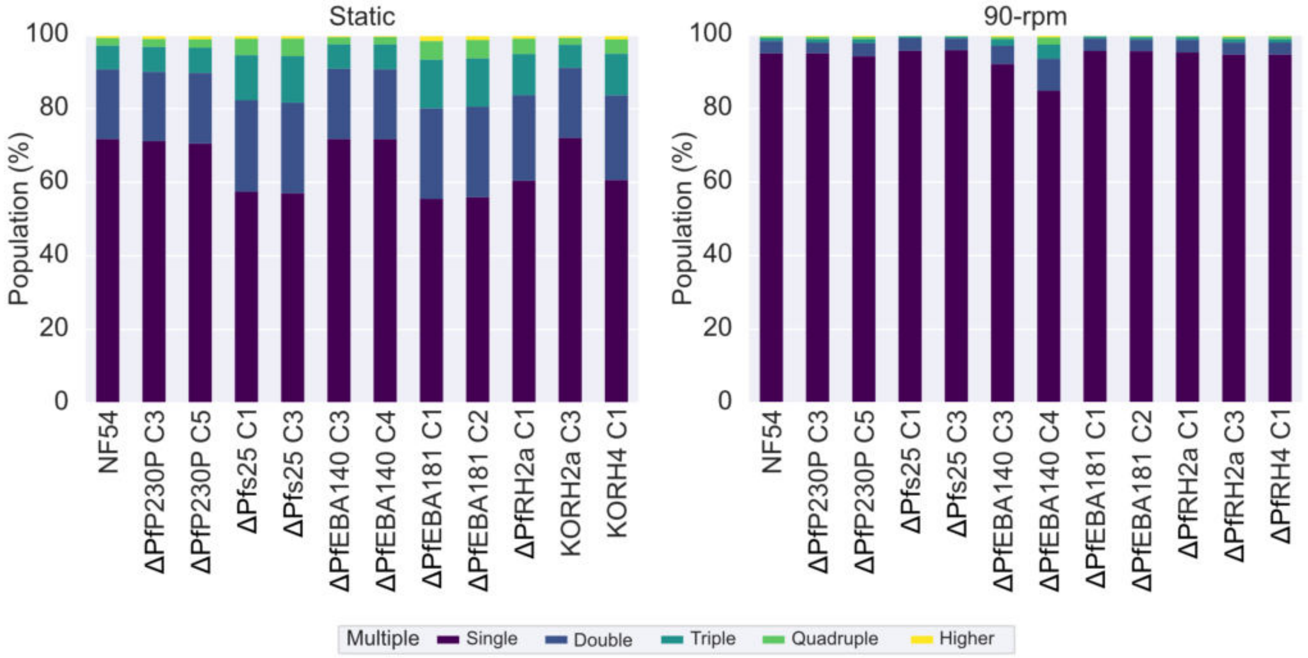

Fig. S8. Investigating the rate of multiple infections of PfEBA and PfRH knockout lines under static and shaking. The percentage of ring-stage-infected erythrocytes (excluding higher DNA counts defined as late-stage) that are made up of multiple infections. The graph represents an average of the peaks from the invasion cycle in which the MEI peaks were clearly defined. There is no data for △PfEBA175 as the peaks of the MEI were not clear in any of the samples for the assay in which it was tested.

Movie S1. Top and side view of 6-well plate with 5 ml of media on an orbital shaker as shaking speed is increased. Available at https://zenodo.org/doi/10.5281/zenodo.13372477.

Movie S2. Top and side view of 50 ml falsk with 5 ml of media on an orbital shaker as shaking speed is increased. Available at https://zenodo.org/doi/10.5281/zenodo.13372477.

Movie S3. Top and side view of 24-well plate with 1 ml of media on an orbital shaker as shaking speed is increased. Available at https://zenodo.org/doi/10.5281/zenodo.13372477.

Movie S4. Top and side view of 96-well plate with 100 ul of media on an orbital shaker as shaking speed is increased. Available at https://zenodo.org/doi/10.5281/zenodo.13372477.

References

1. P Alpresa, S Sherwin, P Weinberg, M Van Reeuwijk, Orbitally shaken shallow fluid layers. II. An improved wall shear stress model. *Phys. Fluids* **30** (2018).
